# Transcranial magnetic stimulation tracks subminute changes in cortical excitability during propofol anesthesia

**DOI:** 10.1002/acn3.50981

**Published:** 2020-02-15

**Authors:** Roman Gersner, Carmen Paredes, Mustafa Q. Hameed, Sameer C. Dhamne, Alvaro Pascual‐Leone, Alexander Rotenberg

**Affiliations:** ^1^ The Neuromodulation Program Department of Neurology Boston Children’s Hospital Harvard Medical School Boston Massachusetts; ^2^ Department of Neurosurgery Boston Children’s Hospital Harvard Medical School Boston Massachusetts; ^3^ Department of Neurology Hebrew SeniorLife Harvard Medical School Boston Massachusetts; ^4^ Institut Guttmann Universitat Autonoma Barcelona Spain; ^5^ Berenson‐Allen Center for Noninvasive Brain Stimulation Department of Neurology Beth Israel Deaconess Medical Center Harvard Medical School Boston Massachusetts

## Abstract

Automated anesthesia systems that continuously monitor cortical excitability (CE) changes to govern drug infusion rates, are desirable. Paired‐pulse transcranial magnetic stimulation (ppTMS), with electromyography (EMG), provides noninvasive CE measures. We tested whether, and with what temporal resolution, ppTMS‐EMG detects dose‐dependent CE in rats anesthetized with continuous intravenous propofol. Motor‐evoked potentials (MEPs) were recorded every 20 seconds as either propofol bolus or change in infusion rate was applied. ppTMS‐derived measures varied in direct proportion to propofol dose with subminute temporal resolution. We conclude that ppTMS‐EMG enables real‐time markers of target engagement by anesthetics that may be incorporated into an automated device.

## Introduction

Transcranial magnetic stimulation (TMS) is a well‐tolerated, noninvasive method for focal cortical stimulation,^1^ where small intracranial electrical currents are induced by a powerful and fluctuating extracranial magnetic field. TMS applied over the motor cortex and coupled with electromyography (TMS‐EMG) is widely used to study cortical plasticity and excitability, by measuring changes in motor‐evoked potentials (MEPs).^2^, ^3^, ^4^, ^5^, ^6^ Paired‐pulse TMS (ppTMS) involves delivering a conditioning stimulus pulse (CS) followed by a test stimulus (TS) after a predetermined interstimulus interval (ISI) to modulate inhibitory or excitatory circuits. A subset of ppTMS protocols, long interval ppTMS (LI‐ppTMS) in rats, as in humans, activate intracortical inhibitory circuits and can detect changes in long‐interval intracortical inhibition (LICI) pursuant to pharmacologic intervention or injury.^7^, ^8^, ^9^

Many general anesthetics shift the excitatory–inhibitory balance of brain signaling toward greater inhibition, in part by modulating GABA receptor signaling.^10^ Propofol, commonly used in clinical practice, is a highly lipophilic agent with a fast onset and short duration of action^11^ via positive modulation of inhibitory function through GABA_A_ receptors.^12^ Although propofol also has inhibitory effects on neuromuscular transmission,^13^ previous studies in rats indicate that the MEP is preserved under propofol anesthesia.^3^ Given the capacity of LI‐ppTMS to measure cortical inhibition, we tested whether LI‐ppTMS can detect subminute changes in cortical excitability modulated by differential dosing of propofol in rats.

## Materials and Methods

### Animals

In total, 52 male Sprague–Dawley rats (250 ± 20 g) were used. Experiments were approved by the Institutional Animal Care and Use Committee at Boston Children’s Hospital (Boston, MA), and in accordance with the NIH Guide for the Care and Use of Laboratory Animals.

### Anesthesia

Rats were anesthetized with isoflurane and placed into a stereotaxic frame. A venous catheter (27G) was inserted into the lateral tail vein. Following intravenous propofol load (10 mg/kg), isoflurane was gradually decreased over 4 min until completely off, while oxygen supplementation continued (1 l/min). Anesthesia was maintained using continuous propofol infusion (1 mg/kg per min unless otherwise specified, see “Experimental Design” section).

### Electromyography (EMG)

MEPs were recorded with monopolar 27G stainless‐steel needle electrodes inserted into the brachioradialis muscle, and a reference electrode positioned distally in the forelimb contralateral to the side stimulated by the TMS coil.^4^, ^14^ EMG was acquired at 40 kHz, band‐pass filtered at 0.3–20 kHz, and amplified 1000x for post hoc analysis.^4^, ^14^

### Transcranial Magnetic Stimulation (TMS)

Focal TMS was delivered to the motor cortex of anesthetized rats through a figure‐of‐eight coil (diameter: outside = 66 mm, inside = 15 mm; Magstim, Wales, UK).^7^, ^14^ TMS intensity was documented as percent machine output (% MO) with 100% corresponding to maximal electrical current conducted through the magnetic coil. Optimal coil position was defined as that with the lowest stimulation intensity required to elicit lateralized MEPs exclusively in the contralateral forelimb.^4^, ^14^, ^15^, ^16^ Stimulator intensity was further adjusted to find motor threshold (MT), defined as the lowest stimulator intensity necessary to elicit MEPs of ≥20 μV peak‐to‐peak amplitude in ≥5 of 10 consecutive trials.^4^, ^14^

### Experimental design

LI‐ppTMS was used to approximate human LICI protocols.^7^, ^8^, ^16^ Baseline was obtained for each rat (100 msec ISI, 120% MT, 15 pulse pairs, one pair/ 20 sec).

*Experiment 1 (propofol bolus):* After baseline, rats were randomized into (1) no bolus (control), (2) 10 mg/kg, or (3) 20 mg/kg; followed by LI‐ppTMS for 15 min.

*Experiment 2 (varied propofol infusion):* After baseline, rats were randomized into (1) no change in infusion rate (1 mg/kg per min; control) or (2) increase to 2 mg/kg per min propofol for 15 min followed by return to 1 mg/kg per min. Each group was monitored by LI‐ppTMS for 30 min.

### Data analysis

All data are presented as mean ± SEM. Peak‐to‐peak voltage of the response to the CS (Response_CS_) and TS (Response_TS_) was measured. Paired‐pulse inhibition was computed as Response_TS_:Response_CS_. All measures were normalized and log transformed. Differences between groups were determined by repeated‐measures ANOVA and Bonferroni post hoc tests, with group as the between‐subject factor and time as the within‐subject factor.

## Results

### Experiment 1: MEP amplitude decreases with increasing doses of propofol bolus

**Figure 1:**
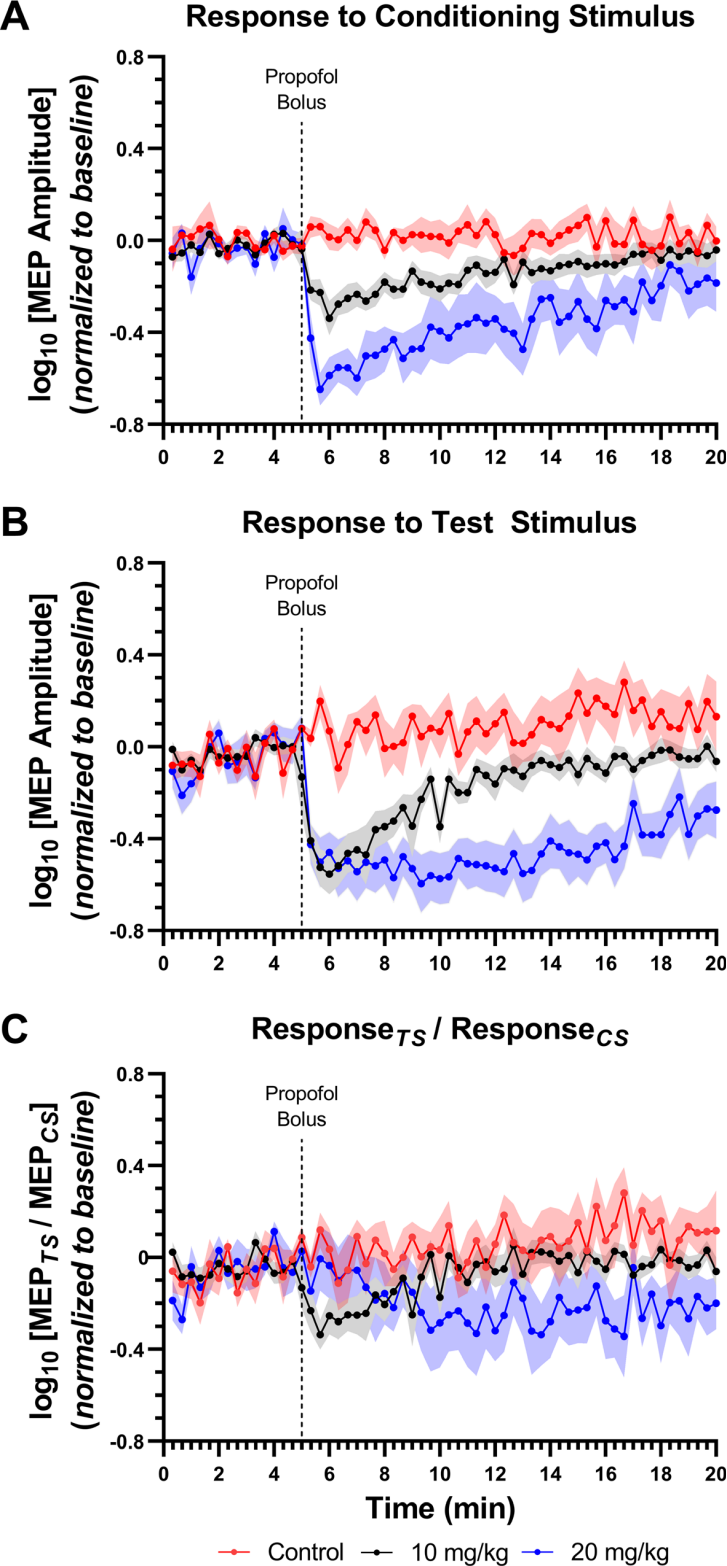
Dose‐dependent decrease in MEP amplitude after propofol bolus. Data are presented as mean ± SEM of MEP amplitude recorded at baseline and at follow‐up timepoints. Following a short baseline, animals received either 10 mg/kg, 20 mg/kg or no bolus of IV propofol. The dotted line indicates time of propofol bolus administration. (A) Propofol significantly reduced the response to the conditioning stimulus in a dose‐dependent manner. (B) However, minimal MEP amplitude in response to the test stimulus was similar following either bolus, with 20 mg/kg resulting in a prolonged decrement. (C) No changes in the inhibition ratio were observed.

Repeated‐measures ANOVA of Response_CS_ amplitudes (Fig. 1A) revealed no significant main effect of group, but a significant main effect of time (*F* (2,44) = 7.63; *P* < 0.001) and interaction between the two (*F* (2, 88) = 2.36; *P* < 0.001). Post hoc analysis revealed that propofol boluses of both 10 mg/kg (*P* < 0.001) and 20 mg/kg (*P* < 0.001) significantly reduced Response_CS_. Moreover, the identified MEP decrease was dose‐dependent (*P* = 0.01), was evident within 20 sec, and significant difference from control lasted for ~7 min after 10 mg/kg and ~12 min after 20 mg/kg boluses.

In Response_TS_ measures (Fig. 1B), significant main effects of group (*F*(2,44) = 36.03; *P* < 0.001), time (*F*(2,44) = 3.40; *P* < 0.001), and interaction (*F*(2,88) = 2.41; *P* < 0.001) between the two factors were observed. Changes in amplitude were evident within 20 sec of the bolus. In rats that received a bolus of 10 mg/kg, the effect lasted for ~5 min before trending toward recovery to baseline. Notably, while minimal Response_TS_ amplitude was similar following either the 10 mg/kg or 20 mg/kg propofol bolus, rats receiving 20 mg/kg showed a prolonged decrement, lasting ~12 min and remaining practically unchanged during the first 8 min of this period. These findings indicate a floor effect for Response_TS_ after the 20 mg/kg bolus. As in the first group, a complete return to baseline was not achieved by the end of the follow‐up.

No significant change in the inhibition ratio was observed (Fig. 1C).

### Experiment 2: MEP amplitude is inversely affected by changing continuous propofol infusion rate

**Figure 2:**
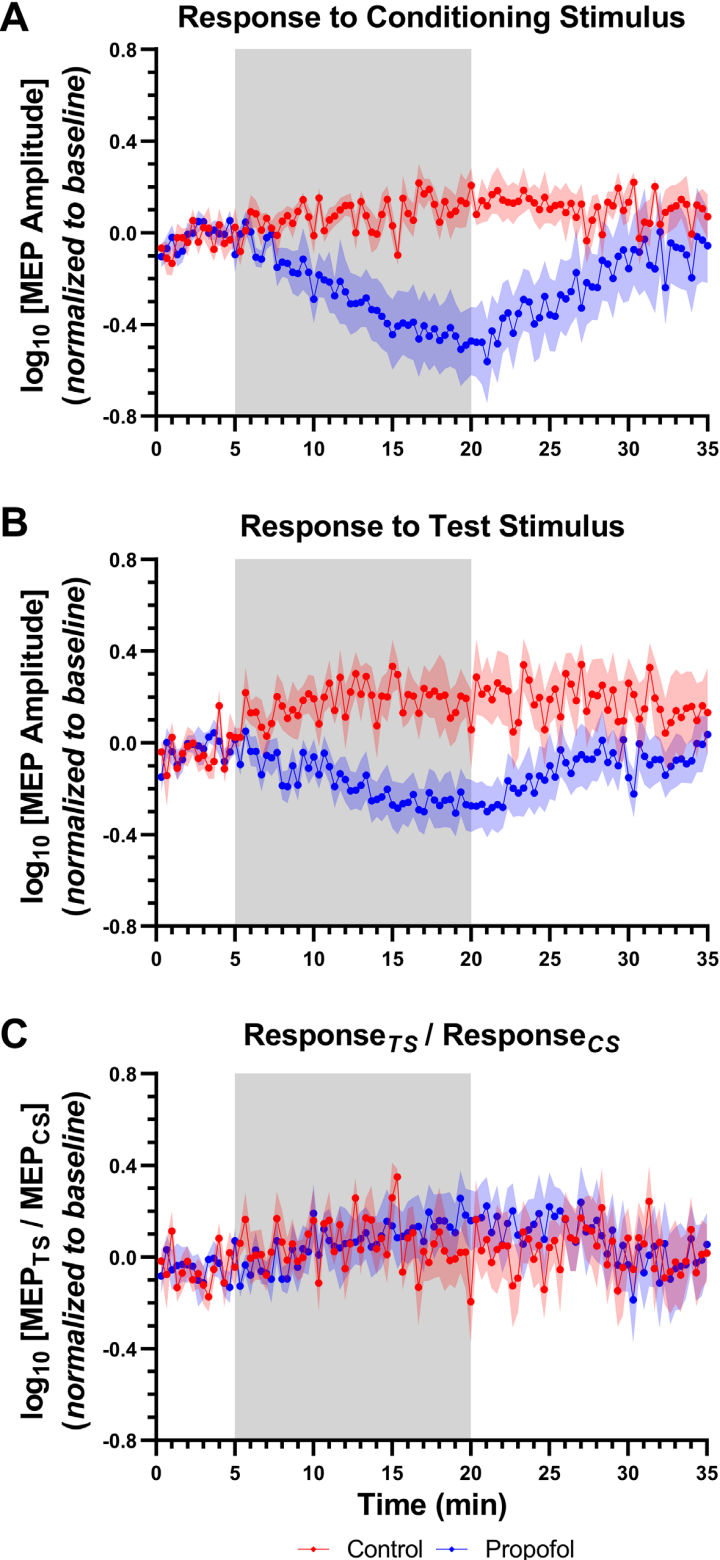
Dose‐dependent decrease in MEP amplitude after propofol bolus. Data are presented as mean ± SEM of MEP amplitude recorded at baseline and at follow‐up timepoints. Following a short baseline, animals received an increased infusion rate of 2 mg/kg per min during 15 min, before returning to 1 mg/kg per min for another 15 min. A control group received a continuous infusion rate of 1 mg/kg per min for the entire 30‐min period. The shaded region indicates the timing of propofol infusion rate changes. (A, B) Increasing the infusion rate from 1 to 2 mg/kg per min resulted in a progressive decrease in the MEP amplitudes in response to the conditioning and test stimuli, respectively. When the infusion rate was restored to 1 mg/kg per min, a progressive increase in both responses was observed. (C) No changes in the inhibition ratio were observed.

In Response_CS_ measures (Fig. 2A), significant main effects of group (*F*(1,89) = 6.27; *P* = 0.026), time (*F*(1,89)=2.83; *P* < 0.001) and interaction between the factors (F(1,89)=2.83; p < 0.001) were found. Increasing the infusion rate (from 1 to 2 mg/kg/min) provoked a progressive decrease in the MEP amplitude, reaching a minimum value 13 min after the rate was increased. With the infusion rate restored to 1 mg/kg/min, a progressive increase in amplitude was observed, reaching baseline levels after 10 min.

In Response_TS_ measures (Fig. 2B), significant main effects of group (*F*(1,89) = 6.62; *P* = 0.023), time (*F*(1,89) = 1.5; *P* = 0.002), and interaction between the factors (*F*(1,89) = 1.5; *P* = 0.002) were observed. A minimum value was reached 10 min following the increase in rate. The amplitude returned to baseline levels 6 min after restoration of infusion rate to 1mg/kg per min.

No significant change in the inhibition ratio was observed (Fig. 2C).

## Discussion

We demonstrate for the first time that propofol modulates a LI‐ppTMS‐EMG measure of cortical excitability parameters in a dose‐dependent manner, and that dose‐dependent fluctuation in inhibition is detectable with subminute temporal resolution. We identified rapid decreases in MEP amplitudes following propofol bolus, and gradual dose‐dependent changes in MEP amplitudes following changes in the propofol infusion rate. Notably, a conspicuous change in the MEP was detected following both CS and TS. These data indicate that individual MEP amplitudes may be valid markers of change in cortical excitability in LI‐ppTMS protocols, in addition to the ratio of MEPs produced by the CS and TS. While the MEP ratio is the typical primary measure in LI‐ppTMS studies, Response_TS_ patterns were like Response_CS_ changes in our experiments, resulting in an essentially stable MEP ratio.

Propofol’s mechanism of action includes both potentiation of GABA_A_ 17 and voltage‐gated sodium channel block.^18^ Standalone GABA_A_ potentiation decreases the Response_TS_:Response_CS_ ratio, while sodium channel blockers reduce MEP amplitude.^18^ Our results indicate that LI‐ppTMS is more sensitive to the latter propofol effect where increasing propofol doses decrease corticospinal excitability and, correspondingly, the MEP amplitude. These alterations of brain excitability were identified within 20 sec following changes in propofol dose, predictably due to the rapid penetration of propofol through the blood‐brain barrier and its fast distribution to the central nervous system.

Recovery to baseline took ~10–12 min from maximal Response_CS_ suppression, which mimics the clinical timeline of recovery from propofol anesthesia.^19^ Together with a decrease in MEP amplitude within 20 sec of bolus administration, this suggests a high temporal resolution of LI‐ppTMS in measuring depth of anesthesia. Our results also suggest that propofol‐induced changes in voltage‐gated sodium channel‐dependent excitability are more readily measured by single‐pulse TMS than GABAergic inhibition assessed by LI‐ppTMS. Moreover, since a floor effect is identified in Response_TS_ in both experiments, the Response_CS_ is a better measure of propofol‐induced anesthesia level. This floor effect also points to follow‐up studies where stimulus strength and inter‐pulse interval may be modified to mitigate the complete Response_CS_ inhibition and thus enable use of the Response_TS_:Response_CS_ ratio.

We aimed to evaluate the feasibility of using LI‐ppTMS‐EMG to detect changes in cortical excitability modulated by propofol anesthesia. However, we note that we assessed only single bolus doses and infusion rate changes per experiment. Other regimens, including multiple consecutive bolus doses, additional infusion rate changes, and tests of other anesthetics warrant future studies. Further, beyond the scope of this report, our results raise prospects for coupling LI‐ppTMS with EEG to measure pharmacologically induced cortical changes excitability outside of the motor cortex.

## Conclusion

We demonstrate for the first time that protocols employing motor cortex TMS coupled with EMG can detect changes in cortical excitability with subminute resolution, which raises prospects for the use of TMS in closed‐loop anesthesia systems. While the MEP ratio is the typical primary measure in LI‐ppTMS studies, our data also indicate that the MEP amplitudes pursuant to both conditioning and test stimuli may be valid markers of changes in intracortical inhibition in LI‐ppTMS protocols, and that the ratio may remain misleadingly stable in the case of similar modulation of individual MEP responses. Beyond the scope of this report, our results raise prospects for coupling LI‐ppTMS with EEG or other readouts to measure cortical excitability outside of the motor cortex.

## Conflict of Interest

A.P.L. serves on the scientific advisory boards for Starlab Neuroscience and Neuroelectrics. A.R. is a founder and advisor to Neuromotion; serves on the scientific advisory board of EpiHunter, Gamify and NeuroRex; and has received consulting fees or research support from Brainsway, Cavion, Neuroelectrics, Roche, Sage, and Takeda, outside the submitted work. The other authors declare no competing interests.
